# Cancer-Associated Fibroblasts Enhance Oxaliplatin Resistance in Colorectal Cancer Cells via Paracrine IL-6: An In Vitro Study

**DOI:** 10.5812/ijpr-160886

**Published:** 2025-07-21

**Authors:** Jiahui Wang, Yanxin Lu, Zhiyong Wang, Pei Wei

**Affiliations:** 1 Department of Immunology, Zhuhai Campus of Zunyi Medical University, Zhuhai, China

**Keywords:** Oxaliplatin, Cancer-Associated Fibroblasts, Interleukin-6, Colorectal Neoplasms

## Abstract

**Background:**

Colorectal cancer (CRC) often develops resistance to oxaliplatin (L-OHP), a key chemotherapeutic agent. Cancer-associated fibroblasts (CAFs) in the tumor microenvironment are implicated in chemoresistance, but their role in L-OHP resistance via interleukin-6 (IL-6) secretion remains unclear.

**Objectives:**

The presnt study investigated how CAFs contribute to L-OHP resistance in CRC, focusing on IL-6 secretion and its impact on cancer cell survival.

**Methods:**

NIH3T3 fibroblasts were co-cultured with murine (CT26) or human (DLD1) colon cancer cells and treated with L-OHP. The supernatant IL-6 levels were measured by enzyme-linked immunosorbent assay (ELISA). Indirect co-culture using Transwell chambers was employed to separate CAF and tumor cell effects. Conditioned media (CM) from both cell types were collected and analyzed for IL-6. Cytotoxicity assays were conducted to assess the survival of L-OHP-treated CT26 cells in the presence of CAF-derived CM, with or without an IL-6-neutralizing antibody.

**Results:**

Co-culture significantly increased IL-6 secretion, which was further amplified by L-OHP. The IL-6 levels in CAF-derived CM were approximately 3.5-fold higher than in tumor cell-derived CM. The CAF-derived CM improved the survival of L-OHP-treated CT26 cells, an effect reversed by IL-6-neutralizing antibodies. Furthermore, adding exogenous IL-6 to tumor cell-derived CM also enhanced survival. Similar IL-6 upregulation in cisplatin-treated CAFs suggests a broader role in platinum-based resistance.

**Conclusions:**

The CAFs promote L-OHP resistance in CRC through IL-6 secretion, enhancing cancer cell survival. Therefore, targeting CAFs and IL-6 signaling may help overcome chemoresistance in CRC.

## 1. Background

Colorectal cancer (CRC) is one of the most common malignancies worldwide, with over 50% of patients diagnosed at an advanced stage (1). In such cases, the prognosis is poor, with a 5-year relative survival rate of approximately 10%, and systemic chemotherapy remains the primary treatment (2). Platinum-based drugs, such as oxaliplatin (L-OHP), are widely used, and their combination with 5-fluorouracil/folinic acid has increased remission rates to over 50% in metastatic CRC, with median survival approaching two years (3). However, nearly all metastatic CRCs eventually develop resistance to L-OHP (4), underscoring the need to understand the mechanisms of chemoresistance.

Chemoresistance results from complex interactions between tumor cells and their microenvironment (5). Among its key players, cancer-associated fibroblasts (CAFs) play a central role in modulating drug resistance and tumor behavior (6). The CAFs have emerged as critical regulators of chemoresistance in CRC through diverse mechanisms, including extracellular matrix remodeling, promotion of cancer stemness, and induction of epithelial-mesenchymal transition (EMT), all of which reduce chemosensitivity (7). Emerging evidence shows that CAFs communicate with cancer cells via extracellular vesicles carrying non-coding RNAs, such as long non-coding RNAs (lncRNAs) and microRNAs (8, 9). These molecules activate oncogenic pathways like β-catenin, suppress apoptosis, and enhance tumor cell survival under chemotherapeutic stress. Additionally, CAFs secrete cytokines and growth factors that remodel the tumor microenvironment and further promote drug resistance.

Among these factors, interleukin-6 (IL-6) plays a pivotal role by activating JAK/STAT3 signaling, which drives EMT, cell proliferation, and survival, thereby contributing to L-OHP resistance (10). Moreover, hypoxic conditions further enhance IL-6 secretion by CAFs through HIF-1α-mediated pathways, amplifying chemoresistance (11). Therefore, the IL-6 axis is a key mediator of CAF-induced chemoresistance in CRC and represents a promising therapeutic target to overcome drug resistance and improve outcomes. Here, we investigated the specific role of CAF-derived IL-6 in mediating L-OHP resistance in CRC cells and explored the underlying molecular mechanisms.

## 2. Objectives

The present study investigated the contribution of CAFs to L-OHP resistance in CRC cells, with a focus on IL-6 secretion and its impact on cancer cell survival.

## 3. Methods

### 3.1. Reagents

The following reagents and materials were used in this study: Oxaliplatin (Abmole, Shanghai, China); cisplatin (Tao Su Biotech Co., Ltd., Shanghai, China); fetal bovine serum (FBS; Inner Mongolia Jinyuankang Biotech, Helingeer, China); mouse and human IL-6 enzyme-linked immunosorbent assay (ELISA) kits (Abcam, Shanghai, China); MTT assay kit (Keygene Biotech, Nanjing, China); and cell culture plates and Transwell chambers (Guangzhou Jet Bio-Filtration, Guangzhou, China).

### 3.2. Cell Culture

Murine fibroblast NIH3T3, murine colon carcinoma CT26, and human colon carcinoma DLD1 cell lines were obtained from the Cell Bank of the Chinese Academy of Sciences (Shanghai, China). Cells were cultured in Dulbecco’s modified Eagle medium (DMEM) with 10% FBS and 1% penicillin/streptomycin at 37°C in 5% CO_2_. Cell lines were authenticated via short tandem repeat profiling, and mycoplasma contamination was tested before each experiment.

### 3.3. Cell Co-culture in Transwell Chambers

CT26 and NIH3T3 cells were co-cultured in Transwell chambers (0.4 µm pore size) to enable paracrine signaling without direct contact. For CT26-derived conditioned medium (CM), CT26 cells (1 × 10^5^/well) were seeded in the bottom wells and NIH3T3 cells (1 × 10^5^/chamber) in the upper chambers. After 24 hours of co-culture with L-OHP, the upper chambers were removed, and CT26 cells were incubated in serum-free medium for 48 hours to collect the supernatant as CT26-derived CM. For NIH3T3-derived CM, the seeding positions were reversed, and the same steps were followed. To control for L-OHP-induced cytotoxicity, cells in the bottom wells were stained with crystal violet after CM collection. Stained cells were solubilized in 2% deoxycholic acid, and absorbance was measured at 595 nm. The CM volumes used for IL-6 ELISA were normalized to viable cell counts.

### 3.4. Enzyme-Linked Immunosorbent Assays

The ELISA kits were used to quantify IL-6 levels in cell culture supernatants according to the manufacturer’s instructions and a previous study (12). Briefly, standards and samples at varying concentrations were added to a 96-well pre-coated plate and incubated for 90 minutes at 37°C. After washing, an HRP-conjugated polyclonal IL-6 antibody was added to each well and incubated for an additional 60 minutes at 37°C. Following another wash step, TMB substrate was added to develop the reaction, which was then stopped with sulfuric acid. Color intensity was measured at 450 nm using a microplate reader (Epoch, BioTek Instruments).

### 3.5. Cytotoxicity Assays

Cytotoxicity was assessed using the MTT assay, as described previously (12). After treatment, 20 µL of MTT solution (5 mg/mL in PBS) was added to each well and incubated for three hours. The plate was then centrifuged at 1800 × g for 5 minutes at 4°C. The supernatant was carefully removed, and 150 µL of buffered DMSO was added to each well, followed by gentle shaking. Absorbance was measured at 570 nm using a microplate reader. Cell viability was expressed as a percentage relative to vehicle-treated controls, which were defined as 100% viable. All assays were performed in triplicate.

### 3.6. Statistical Analysis

All data are presented as the mean ± standard error (SE) from at least three independent experiments. Statistical analyses were conducted using GraphPad Prism 8.0 software. Comparisons between two groups were made using an unpaired two-tailed Student’s *t*-test. For comparisons involving more than two groups, a one-way analysis of variance (ANOVA) followed by Tukey’s post hoc multiple comparison test was performed. P-values < 0.05 were considered statistically significant.

## 4. Results

**Figure 1.**
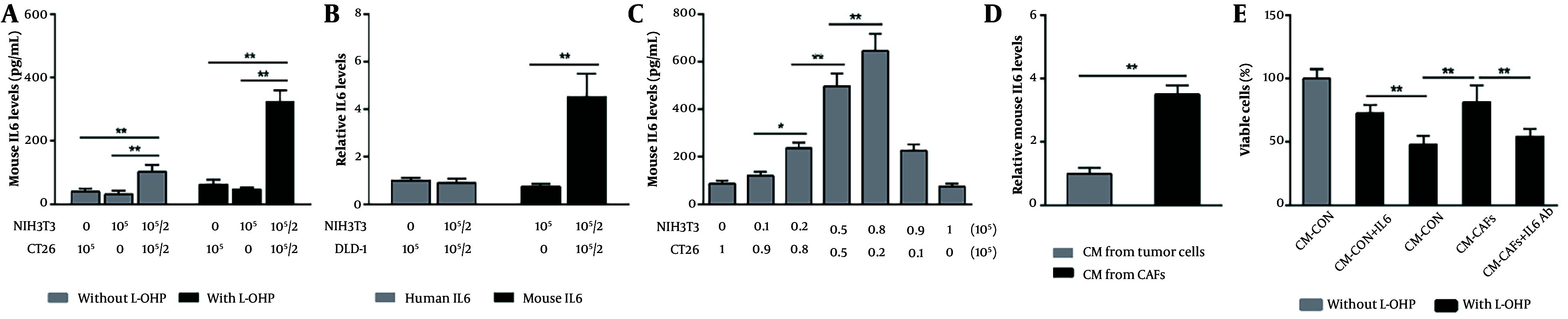
Cancer-associated fibroblasts (CAFs) enhance oxaliplatin (L-OHP) resistance in colorectal cancer (CRC) cells through paracrine interleukin-6 (IL-6). A, mouse IL-6 levels in culture supernatants from individually cultured or co-cultured CT26 and NIH3T3 cells, with or without L-OHP (10 μM) treatment for 36 h, were measured by enzyme-linked immunosorbent assay (ELISA); B, human and mouse IL-6 levels in culture supernatants from co-cultured DLD1 and NIH3T3 cells treated with L-OHP (10 μM) for 36 h were determined by ELISA; C, correlations between IL-6 levels and adjusted inoculation volumes in co-cultured CT26 and NIH3T3 cells treated with L-OHP (10 μM) for 36 h were assessed by ELISA; D, NIH3T3 and CT26 cells were seeded independently in the lower compartments of Transwell chambers and co-incubated without direct contact in the presence of L-OHP (10 μM) for 24 h. After removing the upper chamber cells and drug-containing medium, the lower chamber cells were reincubated in fresh medium for 48 h to collect distinct conditioned media (CM). Mouse IL-6 levels in CM were measured by ELISA and normalized to cell number; E, the effects of different CM supplemented with an IL-6 neutralizing antibody (10 μg/mL) or exogenous mouse IL-6 (1 ng/mL) on CT26 cell viability after L-OHP (10 μM) treatment for 72 h were evaluated by MTT assay. * P < 0.05, ** P < 0.01.

NIH3T3 cells have been shown to transition between fibroblasts and CAFs (13, 14). To investigate the role of CAFs in L-OHP resistance, we co-cultured NIH3T3 fibroblasts with murine colon cancer cells (CT26) and treated them with L-OHP for 36 hours. The ELISA analysis revealed that, without L-OHP, the IL-6 level in the co-culture supernatant was 2.4- and 2.9-fold higher than in supernatants from cancer cells or fibroblasts cultured alone, respectively (Figure 1A). Following L-OHP exposure, IL-6 levels in the co-culture supernatant increased further, reaching 5.2- and 6.8-fold higher than in monocultured cancer cells and fibroblasts, respectively (Figure 1A). These results suggest that fibroblast-cancer cell co-culture enhances IL-6 secretion, which is further amplified by L-OHP.

To determine the source of IL-6, we considered prior findings that NIH3T3 cells can be induced into CAFs even when co-cultured with human-derived tumor cells, despite species differences (15, 16). We co-cultured NIH3T3 with human colon cancer cells (DLD1) in the presence of L-OHP and measured mouse and human IL-6 levels. Mouse IL-6 levels increased predominantly in the supernatant (Figure 1B). Additionally, by varying the co-culture ratios of NIH3T3 and CT26 cells, we observed that IL-6 concentration rose with increasing NIH3T3 cell density (Figure 1C). In contrast, CT26 cell density had only a minor effect on supernatant IL-6 levels (Figure 1C), indicating that CAFs are the primary source of elevated IL-6.

To minimize potential inaccuracies from L-OHP-induced cell death, we used Transwell chambers to co-culture NIH3T3 and CT26 cells without direct contact. Each cell type was seeded separately in the lower compartments and incubated together under L-OHP exposure. After 24 hours of L-OHP treatment, the upper chamber cells and drug-containing medium were removed, and the lower chamber cells were re-incubated in fresh medium. This method allowed a separate collection of CM from CAFs and tumor cells. Following an additional 48-hour incubation, CM was harvested for IL-6 analysis and normalized by cell number. We found that IL-6 levels in CAF-derived CM were approximately 3.5-fold higher than in tumor cell-derived CM (Figure 1D), indicating that IL-6 was predominantly secreted by CAFs.

Moreover, cytotoxicity assay results demonstrated that CAF-derived CM enhanced the survival of L-OHP-treated CT26 cells, an effect reversed by IL-6 neutralizing antibody (Figure 1E). Conversely, supplementing tumor cell-derived CM with exogenous IL-6 significantly increased CT26 cell survival under L-OHP treatment (Figure 1E). These results suggest that IL-6-mediated L-OHP resistance is driven mainly by CAFs rather than tumor cells. Notably, we observed a similar IL-6 secretion pattern in CAFs exposed to cisplatin, another platinum-based drug, strongly suggesting a broader mechanism of platinum resistance in colon cancer (data not shown).

## 5. Discussion

Our findings demonstrate that CAFs critically contribute to L-OHP resistance in CRC, largely by modulating the tumor microenvironment through IL-6 secretion. These results align with prior studies implicating CAFs in chemoresistance via cytokine and factor secretion (7). The observed increase in IL-6 secretion in co-culture systems underscores the importance of tumor-stroma interactions in driving chemoresistance. The significantly higher IL-6 levels in CAF-derived CM compared to tumor cell-derived CM suggest that CAFs are a major source of IL-6 in the tumor microenvironment. This finding aligns with growing evidence implicating CAFs in regulating cytokine networks that promote tumor progression and drug resistance. For example, studies show that CAFs secrete IL-6 and IL-11 to activate STAT3 signaling in cancer cells, enhancing survival and resistance to apoptosis (17).

Our results extend these findings by demonstrating that CAF-derived IL-6 directly increases the survival of L-OHP-treated CRC cells, an effect reversible with IL-6-neutralizing antibodies. The use of Transwell chambers allowed us to isolate the contributions of CAFs and tumor cells to IL-6 secretion, clarifying their respective roles. The reversal of CAF-mediated survival advantages by IL-6 blockade highlights the functional significance of IL-6 in this process. Furthermore, exogenous IL-6 enhanced the survival of L-OHP-treated tumor cells, suggesting that IL-6 signaling is a key mechanism by which CAFs promote chemoresistance. These findings are supported by previous studies showing that IL-6 activates downstream pathways such as STAT3 and MAPK, which are known to confer chemoresistance (17, 18).

Recent studies have elucidated molecular mechanisms regulating IL-6 secretion by CAFs, contributing to chemoresistance and tumor progression. The CAFs utilize secretory autophagy to stimulate IL-6 release, with the secreted IL-6 subsequently promoting the development of chemotherapy resistance (19). Additionally, CAF-derived IL-6 activates the JAK2/STAT3 pathway in cancer cells, inducing EMT and enhancing metastatic potential, thereby reinforcing a positive feedback loop that sustains IL-6 expression (20). Metabolic regulators such as CPT1C in CAFs also promote IL-6 secretion, inducing an immunosuppressive M2-like macrophage phenotype and supporting tumor immune evasion (21). Moreover, hypoxic conditions in the tumor microenvironment upregulate IL-6 secretion by CAFs via HIF-1α-dependent mechanisms, promoting CRC cell proliferation and survival (22). Notably, CAF-tumor cell cross-talk synergistically amplifies IL-6 production, establishing a complex paracrine network essential for chemoresistance and tumor progression (23). Therefore, these insights highlight the multifaceted regulation of IL-6 secretion by CAFs and suggest that targeting these pathways could be a promising strategy to overcome chemoresistance in CRC.

Interestingly, we observed a similar IL-6 secretion pattern in CAFs exposed to cisplatin, suggesting CAFs may broadly contribute to platinum drug resistance. This finding implies that targeting CAFs or their signaling pathways could help overcome resistance to multiple platinum-based therapies. For instance, recent studies have explored small-molecule inhibitors against IL-6 signaling or CAF-specific markers as potential therapeutic strategies (24, 25). Our results support further investigation of these approaches in CRC.

However, our study has several limitations. First, we used a mouse fibroblast cell line rather than primary human cancer-associated fibroblasts (hCAFs). While this model provides a consistent platform to study IL-6-mediated chemoresistance in vitro, it lacks the patient-derived heterogeneity of hCAFs in the human CRC microenvironment. Species differences and cell line phenotypes mean our findings — particularly regarding IL-6 — should be interpreted cautiously for clinical relevance. Future studies using primary hCAFs are needed to validate these results in a more clinically representative setting.

Second, our experimental model employed murine fibroblasts (NIH3T3) co-cultured with human CRC cells (DLD1), creating a heterologous system. Although useful for mechanistic studies and overcoming some technical challenges, this setup does not fully replicate the complexity of the human tumor microenvironment. Species-specific variations in signaling pathways, cytokine profiles, and cell-cell interactions may affect the observed outcomes, potentially limiting direct translational relevance.

Third, while we focused on IL-6, CAFs secrete diverse cytokines and growth factors (e.g., TGF-β, VEGF) that may also contribute to chemoresistance (26, 27). Investigating these additional factors and their interplay with IL-6 could provide a more comprehensive understanding of resistance mechanisms. Moreover, the dynamic tumor microenvironment means CAF-tumor interactions may evolve over time. For example, shifts in cellular states or the presence of other immune cells, such as tumor-associated macrophages (TAMs) or myeloid-derived suppressor cells (MDSCs), could alter cytokine secretion and drug sensitivity (28, 29). Therefore, future studies should explore the temporal and spatial dynamics of CAF-tumor interactions during chemotherapy, leveraging single-cell RNA sequencing and spatial transcriptomics for higher-resolution insights (30, 31).

### 5.1. Conclusions

In summary, our findings demonstrate that CAFs promote L-OHP resistance in CRC through IL-6 secretion, fostering a protective tumor microenvironment that enhances cancer cell survival under chemotherapy. These results underscore the potential of targeting CAFs and IL-6 signaling to overcome chemoresistance in CRC. Further research should explore the broader role of CAF-mediated cytokine networks in drug resistance.

## Data Availability

The dataset presented in the study is available on request from the corresponding author during submission or after publication.
